# The effect of blue light exposure in an ocular melanoma animal model

**DOI:** 10.1186/1756-9966-28-48

**Published:** 2009-04-07

**Authors:** Sebastian Di Cesare, Shawn Maloney, Bruno F Fernandes, Claudia Martins, Jean-Claude Marshall, Emilia Antecka, Alexandre N Odashiro, William W Dawson, Miguel N Burnier

**Affiliations:** 1 The Henry C Witelson Ophthalmic Pathology Laboratory and Registry, McGill University Health Center, Montreal, PQ, Canada; 2 Department of Ophthalmology, University of Florida, Gainesville, Fl, USA

## Abstract

**Background:**

Uveal melanoma (UM) cell lines, when exposed to blue light in vitro, show a significant increase in proliferation. In order to determine if similar effects could be seen in vivo, we investigated the effect of blue light exposure in a xenograft animal model of UM.

**Methods:**

Twenty New Zealand albino rabbits were injected with 1.0 × 10^6 ^human UM cells (92.1) in the suprachoroidal space of the right eye. Animals were equally divided into two groups; the experimental group was exposed to blue light, while the control group was protected from blue light exposure. The eyes were enucleated after sacrifice and the proliferation rates of the re-cultured tumor cells were assessed using a Sulforhodamine-B assay. Cells were re-cultured for 1 passage only in order to maintain any in vivo cellular changes. Furthermore, Proliferating Cell Nuclear Antigen (PCNA) protein expression was used to ascertain differences in cellular proliferation between both groups in formalin-fixed, paraffin-embedded eyes (FFPE).

**Results:**

Blue light exposure led to a statistically significant increase in proliferation for cell lines derived from intraocular tumors (p < 0.01). PCNA expression was significantly higher in the FFPE blue light treated group when compared to controls (p = 0.0096).

**Conclusion:**

There is an increasing amount of data suggesting that blue light exposure may influence the progression of UM. Our results support this notion and warrant further studies to evaluate the ability of blue light filtering lenses to slow disease progression in UM patients.

## Background

Uveal Melanoma (UM) is the most common primary malignant intraocular tumor in adults [1]. The incidence rate for UM ranges from 4.3–10.9 cases per million, depending on the specific criteria used to diagnose this disease [2]. Although it is a relatively uncommon malignancy, approximately 50% of all patients initially diagnosed with UM will end up developing liver metastasis within 10–15 years [3]. Predispositions to this disease include the presence of choroidal nevi, which occur quite frequently within the aging population.

With age, the human lens becomes progressively more yellow. This process is thought to effectively filter more blue light from passing through the yellowed lens [4,5]. Following cataract surgery, the removal of the aged lens is accompanied by loss of natural ability to filter blue light (500-444 nm, The CIE International Diagram for Blue Ranges).

Further studies have suggested that blue light exposure may play a role in the malignant transformation of melanocytes, which can eventually lead to the development of melanoma [6]. It has been previously shown that rats subjected to long-term blue light exposure developed intraocular masses that were pathologically diagnosed as ocular melanoma [7]. A recent statistical study has demonstrated an increased risk of developing dysplastic skin nevi in children previously treated with neonatal blue-light therapy at birth [8]. Several well-documented risk factors for the development of UM have been identified, including age, iris color and skin pigmentation [2]. Even though sunlight exposure is considered a significant risk factor by some [9], the relationship between sunlight exposure and UM development remains controversial [10].

It has been demonstrated in primates that blue light can mediate the production of reactive oxygen species (ROS) in the posterior segment of the eye. This ROS production due to blue light exposure could be responsible for cellular damage to the retinal pigment epithelial (RPE) cells [11]. The production of these ROS may therefore play an important role in the development of age-related macular degeneration [12].

Our laboratory has previously shown that the proliferation rates of human uveal melanoma cell lines increase significantly in vitro after exposure to relatively high amounts of blue light [6]. We therefore propose to extend these preliminary in vitro studies to investigate the potential effects of blue light in an in vivo ocular melanoma animal model [13].

## Methods

The animal model was carried out in compliance with the Association for Research in Vision and Ophthalmology Statement for the Use of Animals in Ophthalmic and Vision Research. The approval of both the Animal Care Committee and the Ethics Subcommittee at McGill University was obtained prior to all experiments.

### Animals

Twenty female New Zealand albino rabbits (Charles River Canada, St-Constant, Québec) were randomly divided into two groups, control and experimental, with mean initial weights of 3.2 ± 0.18 kg and 3.2 ± 0.17 kg respectively. Female animals were used to avoid aggressive conflicts that can occur when group-housing male animals. The animals were immunosuppressed daily using intramuscular injections of cyclosporine A (CsA; Sandimmune 50 mg/ml, Novartis Pharmaceuticals Canada Inc., Dorval, Québec, Canada) in order to avoid rejection of the human cells. CsA administration was maintained throughout the 8-week experiment to prevent tumor regression. The dosage schedule recommended in previous studies was employed: 15 mg/kg/day, 3 days before cell inoculation and during 4 weeks thereafter, followed by 10 mg/kg/day during the last 4 weeks of the experiment [13]. CsA doses were adjusted weekly according to the animal weight to compensate for any weight loss during the experiment.

### Cell line and cell injection procedure

The injection procedure and subsequent animal handling were carried out as previously described [13]. The 92.1 primary human uveal melanoma cell line [14], kindly provided by Dr. Antonia Saornil from the Instituto Universitario de Oftalmobiología Aplicada (IOBA), University of Valladolid, was used. This selection was based on previous studies performed in our laboratory where this cell line demonstrated high proliferative and invasive potential in vitro [15]. The cells were maintained at 37°C in a humidified 5% CO_2_-enriched atmosphere (Thermo Forma Series II Water Jacketed CO_2 _Incubator, Fisher Scientific Limited, Ontario, Canada). The cells were cultured in RPMI-1640 medium (Invitrogen, Burlington, Ontario, Canada), supplemented with 5% heat inactivated fetal bovine serum (FBS; Invitrogen), 1% fungizone (Invitrogen), and 1% penicillin-streptomycin (Invitrogen). One million cells (cellular viability greater than 99%) suspended in 0.1 ml of RPMI-1640 media were injected into the suprachoroidal space of the right eye of each rabbit according to a previously described technique [13]. Ketamine (35 mg/kg; Vetalar, Vetrepharm Canada Inc., Belleville, Ontario, Canada) and xylazine (5 mg/kg; Anased, Novopharm Limited, Toronto, Ontario, Canada) were used as anesthetics during the surgical procedure.

### Blue Light Exposure

The 20 rabbits used in this experiment were randomly divided into two separate groups of 10 rabbits each. The experimental group was exposed to blue light 8 hours per day for the duration of the 8-week experiment. The animals were group-housed in a large pen into which the blue light-emitting apparatus was placed. The apparatus consisted of a large metal cage in which twenty-four 6600 k bulbs were suspended, each covered by a sheet of co-extruded polycarbonate film (Rosco, Color Filter #74 Night Blue) that allowed light only in the blue portion of the spectrum to pass through. This apparatus was placed in the middle of the pen, with suspended bulbs reaching to approximately 6" from the ground to achieve maximal light exposure at eye level. Additionally, the pen was lined with 3' high reflective aluminum to ensure adequate blue light exposure in all areas of the pen. As a rabbit's gaze is typically 10 to 15 degrees below the horizontal plane, 3' high reflective aluminum was adequate to ensure continuous blue light exposure in the direction of gaze. All lights were connected to a timer that turned on at 11 am and turned off at 7 pm daily. Protective goggles were provided to all personnel entering the housing area during the period of blue light exposure. The control group was in the adjacent pen, which was covered by a polycarbonate film (Rosco, Color Filter #15 Deep Straw) that ensured proper blockage of any light within the blue portion of the visible spectrum (500-444 nm, CIE International Diagram for blue light ranges) from entering the control pen.

### Fundoscopy

Indirect ophthalmoscopy of dilated pupils using Tropicamide (Alcon Canada Inc., Mississauga, Canada; Mydriacyl, Alcon Canada Inc.) was performed before cell inoculation to rule out any existing ocular pathologies, and weekly after cell inoculation to clinically document intraocular tumor development.

### Euthanasia

In order to document the time-course of the disease, particularly the development of metastasis, one animal per group was euthanized per week starting at two weeks post-inoculation of cells into the eye. The selection criterion was based on the appearance of the animal, signs of CsA toxicity and veterinary recommendations. The remaining rabbits (n = 4) were sacrificed at the end of the experiment. The method of euthanasia was exsanguination by cardiac puncture following anesthesia using intramuscular ketamine-xylazine (35 mg/kg-5 mg/kg). An autopsy was performed on every animal that was sacrificed. The enucleated eyes and other organs with possible metastatic disease such as lungs, livers and kidneys were collected, macroscopically examined and preserved in 10% phosphate buffered formalin. Formalin-fixed, paraffin-embedded sections of the collected specimens were stained with hematoxylin and eosin for histopathologic assessment.

### Re-Culturing of Cells Post-Euthanasia

The right eye of each rabbit was processed prior to formalin fixation in order to acquire a fresh tumor sample from each rabbit. Cells were cultured in a 6-well plate in 5% FBS supplemented RPMI and grown to confluence before seeding for proliferation assay experiments. All blood collected from cardiac puncture of rabbits during euthanasia was processed via the Ficoll-Paque™ Plus Method (Amersham Biosciences) in order to harvest and culture the buffy coat. This was done in order to capture and document presence of circulating malignant cells (CMCs) throughout the duration of the experiment. CMCs were allowed to adhere to the bottom of the 6-well plate, while remaining non-adherent white blood cells were washed off during subsequent media changes. CMCs were allowed to grow to confluence prior to seeding the proliferation assays. All re-cultured cells (primary tumors, CMCs) were passaged only once in order to maintain any phenotypic changes these cells may have acquired in vivo.

### Immunohistochemistry

Immunohistochemistry was performed using the Ventana BenchMark fully automated machine. The fully automated processing of bar code labeled slides included baking of the slides, solvent-free deparaffinization, and CC1 (Tris/EDTA buffer pH 8.0) antigen retrieval. Slides were incubated with a mouse monoclonal anti-human Proliferating Cell Nuclear Antigen (PCNA) antibody (dilution 1:200; Dako Canada Inc., Mississauga, Ontario; Clone PC10) for 30 min. at 37°C, followed by application of biotinylated secondary antibody (8 min. at 37°C) and an avidin/streptavidin enzyme conjugate complex (8 min at 37°C). Finally, the antibody was detected using the Fast Red chromogenic substrate and counterstained with hematoxylin. As positive controls, sections of human small intestine and colon were used for the PCNA antibody. For negative controls the primary antibody was omitted. Sections were analyzed for PCNA nuclear expression in tumor samples and surrounding ocular tissues. A total of 10 rabbit xenograft (92.1) UMs were used for this analysis. Samples were also independently graded as either positive or negative for PCNA nuclear expression in each of the samples by two different pathologists. The percentage and intensity of overall tumor positivity were also assessed.

### Immunocytochemistry

Cytopsins of all re-cultured cells (primary tumor, CMCs) were made using a Cytospin3 machine (Shandon). Cells from culture were diluted to a concentration of 250,000 cells/ml, and a 300 μL solution at that concentration was placed in each spin to be evenly distributed on each slide. All slides were then immunostained with a primary anti-human mouse monoclonal antibody against Melanosome (Dako Canada Inc., Mississauga, Ontario; Clone HMB-45) using the Ventana™ automated immunostaining machine programmed to use a standard Avidin-Biotin Complex method. HMB-45 is a well-established marker used by pathologists in order to identify the presence of uveal melanoma cells [16,17]. These stainings were done in order to ensure that the re-cultured cells were actually uveal melanoma cells.

### Proliferation Assay

The Sulforhodamine-B based assay kit (TOX-6, Sigma-Aldrich, St. Louis, Missouri, USA) was performed according to the National Cancer Institute protocol [18]. Re-cultured cells obtained from the rabbits (primary tumor, CMCs) were seeded in a 96-well plate at a concentration of 2.5 × 10^3 ^cells per well, with six wells per cell line from each group (blue light, control). Cells were allowed to adhere overnight and incubate for 48 and 72 hours. Following both the 48 and 72 hour incubation periods, cells were fixed to the bottom of the wells using a solution of 50% Trichloroacetic acid (TCA) for 1 hour at 4°C. Plates were then rinsed with distilled water to remove the TCA and excess media and were air-dried. The Sulforhodamine-B dye solution was then added to each well and allowed to stain for 30 minutes. The Sulforhodamine-B solution was subsequently removed by washing with a 1% acetic acid solution and once more allowed to air dry. The dye that had become incorporated into the fixed cells at the bottom of the wells was solubilized in a 10 mM solution of Tris base solution. The absorbance of the solute was measured using a microplate reader at a wavelength of 565 nm.

### Statistical Analysis

Results from the proliferation assays for both time points (48 h, 72 h) were analyzed using the Student's t-test. A result was considered significant when a p-value of < 0.05 was obtained for each t-test performed. Results from the PCNA staining were interpreted using a Correlation analysis. A correlation was drawn by comparing PCNA staining intensity with exposed or non-exposed rabbits. A result was considered significant when a p-value of < 0.05 was obtained.

## Results

### Fundoscopy

At the first week timepoint, 2 animals from the control group and 3 animals from the experimental group had fundoscopically detectable intraocular masses. By week 3, the total number of visible tumors was 5 and 4 in the control and experimental groups, respectively. These numbers remained unchanged until the end of the experiment.

### Histopathological Studies

**Figure 1:**
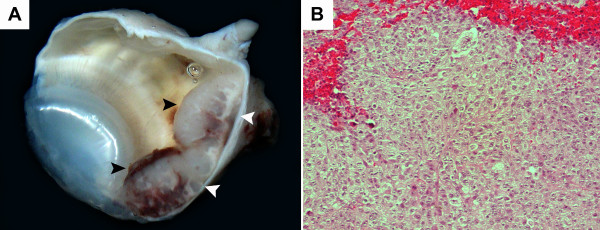
**Gross & histopathological images of an enucleated rabbit eye**. A) Cross section of the right eye (O.D) from a control group rabbit, displaying a large intraocular mass and hemorrhage, at week 5 of the experiment. B) Photomicrograph of the same rabbit eye (O.D), H&E displaying hemorrhage surrounding the tumor cells (200×).

Macroscopically detectable intraocular masses were seen in 6 animals of the control group and 4 animals in the experimental group (Figure 1). Histopathological evaluation of the enucleated eyes revealed tumors in 7 of the animals in the control group and in 5 of the experimental group.

No macroscopic metastatic disease was found in either group. Serial sections of the animals' lungs revealed metastatic disease in 4 animals in the control group and in 4 animals in the experimental group. No liver metastasis was seen. The differences seen between the two groups were not statistically significant.

### Re-Culturing of Cells Post-Euthanasia

A total of 5 primary tumors from the control group and 4 primary tumors from the experimental group were successfully re-cultured (1 passage) for subsequent use in the cytospin analysis and proliferation assays. In addition, 2 CMC cultures from the control group and 1 from the experimental group were retrieved for subsequent cytospin and proliferation assay analysis.

### Immunohistochemistry

**Figure 2:**
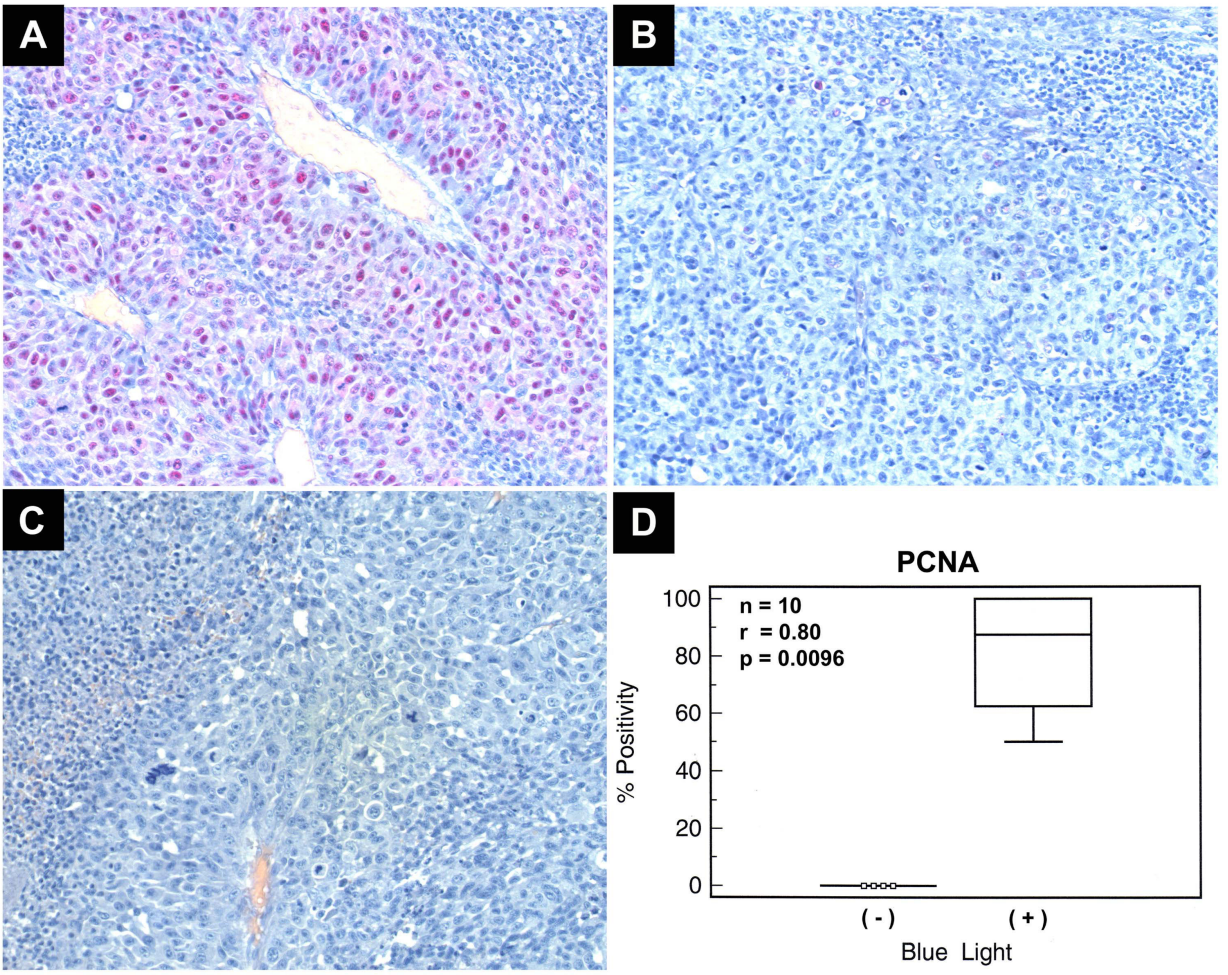
**PCNA Immunostaining comparing FFPE blue light exposed rabbit eyes to control eyes (O.D)**. A) Positive nuclear staining for PCNA in cells (92.1) from a rabbit in the blue light treated group (200×). B) Negative nuclear staining for PCNA in cells (92.1) from a rabbit in the control group (200×). C) Negative Control (200×). D) Box and Whisker plot depicting the relative percentage of PCNA positivity between rabbits exposed to blue light, and those not exposed.

All of the FFPE control rabbit eyes were negative for PCNA (n = 5). The FFPE blue light treated group had 3 rabbit eyes that were highly positive (85–100%), and 2 rabbit eyes that had mild positivity when stained with PCNA (n = 5). A Correlation analysis was preformed to relate staining intensity and blue light exposure. Statistically significant results were obtained (n = 10, r = 0.8, p = 0.0096) (Figure 2).

### Immunocytochemistry

**Figure 3:**
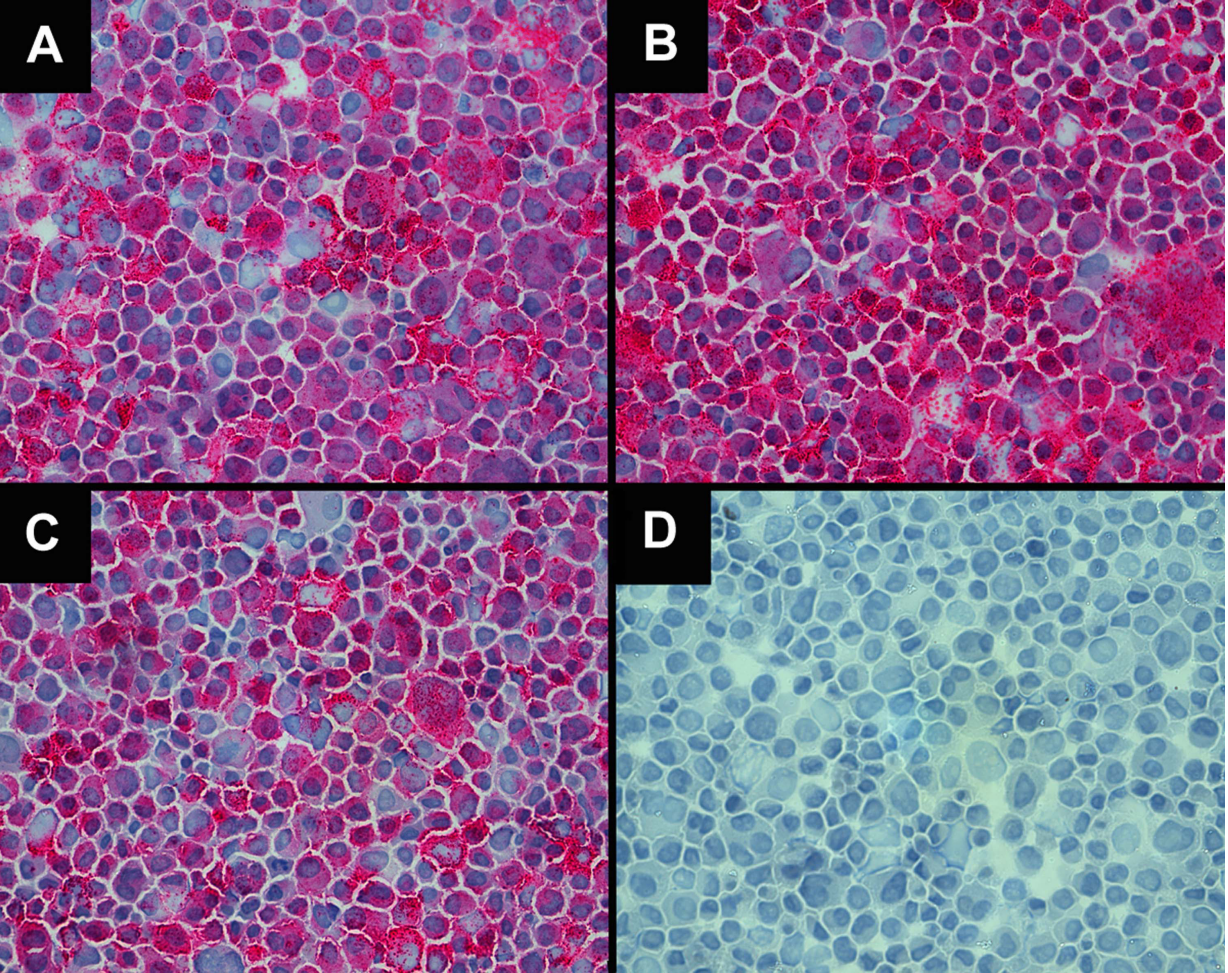
**Cytospins prepared from re-cultred 92.1 cells from rabbit eyes (OD) stained for HMB-45**. A) Cytospin of UM cells (92.1) isolated from the right eye of a control group rabbit. B) Cytospin of UM cells (92.1) isolated from the right eye of a blue light treated rabbit. C) Cytospins of CMCs (92.1) isolated from the blood (buffy coat) of a control group rabbit. D) Negative Control (92.1) (400×).

All re-cultured samples (primary tumors, CMCs) stained positive for the monoclonal mouse anti-human Melanosome marker (Figure 3). This specific positivity indicates that all re-cultured cells used in the proliferation assays were indeed the human uveal melanoma cell line 92.1 that was initially inoculated in the eyes of the rabbits.

### Proliferation Assay

**Figure 4:**
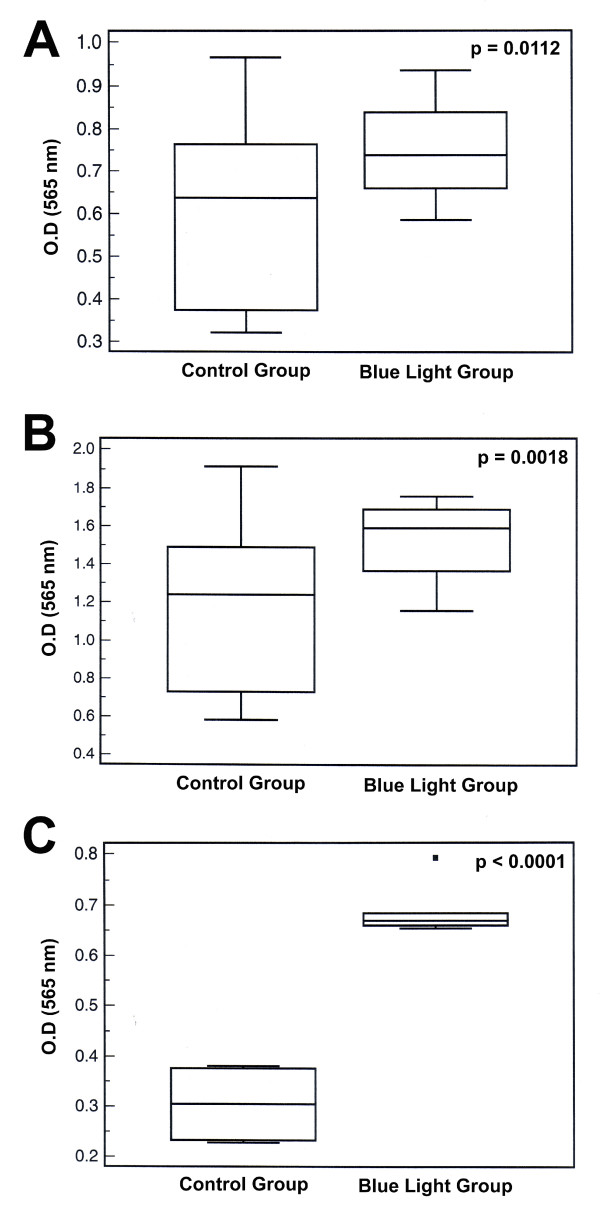
**Box and Whisker plots depicting the change in cellular proliferation of re-cultured 92.1 cells from rabbit eyes (O.D) when exposed to blue light**. A) Change in cellular proliferation of primary tumors after 48 h incubation. B) Change in cellular proliferation of primary tumors after 72 h incubation. C) Change in cellular proliferation of isolated CMCs after 48 h incubation.

Cells from the blue light treated group proliferated significantly faster than the control group cells at the 48 h (p = 0.0112) and 72 h (p = 0.0018) time points. The CMCs isolated from the blue light group proliferated significantly faster (48 h) than the cells from the control group (p < 0.0001) (Figure 4).

## Discussion

Current hypotheses indicate that several environmental and genetic factors may play a role in the progression of uveal melanoma formation [19-21]. Typical phenotypic progression of this disease usually begins with the appearance of benign nevi. Later events include the transformation of the cells within the nevi to a spindle-cell and eventually epithelioid-cell uveal melanoma. Epithelioid cells are considered the most aggressive type of uveal melanoma cells and carry the worst prognosis. This generalized progression towards a more malignant phenotype may also be influenced by exposure to natural sunlight, particularly the UV and blue light portions of the electromagnetic spectrum [22]. A recent meta-analysis by Shah et al identified welding, which is a significant source of blue-light, as a risk-factor for uveal melanoma [20]. Interestingly, ocular melanoma could also be induced by exposing rats to blue-light during an experimental animal model [7].

The rationale behind a possible relationship between blue light and tumorigenesis is that visible light of short wavelengths can cause DNA damage [11]. The secondary mutation can be transferred to further generations of transformed cells ultimately generating a malignant clone. Previous work in our laboratory has shown that blue light increases the proliferation rate of uveal melanoma cell lines [6]. These results also indicated that the use of UV and blue light filtering intra-ocular lenses (IOLs) conferred a protective effect. These IOLs significantly reduced the proliferative effect that blue light caused in the un-protected uveal melanoma cells. As in vitro results can not necessarily be extrapolated to understand in vivo effects, we performed the current experiment using an established animal model of uveal melanoma [13]. When the re-cultured cells from the experimental group were compared to the control group, higher proliferation rates were seen. In other words, the blue light was able to penetrate to the posterior of the eye and induce the necessary molecular changes that ultimately resulted in higher proliferation rates of uveal melanoma cells. Similarly, the PCNA staining confirms these findings by being significantly more expressed in the blue light treated group when compared to controls.

A question that one may raise is whether or not the changes secondary to blue-light exposure are permanent. We have reasons to believe that they are. The fact that even the CMCs from the experimental group presented with higher proliferation rates is further evidence that the changes induced by blue light exposure are not transient. Whatever molecular changes were induced, the secondary generations of those cells still exhibited a higher proliferation profile, even after being in circulation and away from a blue light source. The number of eyes that developed tumors, primary tumor size and number of metastasis were not statistically different between groups. We believe that the difference in proliferation rate was not significant enough to cause measurable differences in tumor size during the time period of the study.

Another important question to be answered is whether blue light can induce malignant transformation of a normal melanocyte. The main barrier to get this answer is the scarcity of established cell lines of normal uveal melanocytes. Even if development and availability of such cell lines were adequate, there would likely be numerous changes in gene expression profiles after successive passages and immortalisation, rendering any conclusions drawn from such a comparison incomplete. However, there are a number of epidemiological studies on pediatric literature showing clinical evidence that blue light can indeed affect normal melanocytes. Neonates exposed to blue light phototherapy as a treatment for jaundice present with a larger number of dysplastic cutaneous nevi later in life [23]. Nevi count tends to be higher and the average nevus size is also larger in the exposed group compared to controls [24]. Considering that dysplastic nevus is the most important predisposing lesion for cutaneous melanoma, this is strong evidence that blue-light can induce the transformation of a normal melanocyte into a pre-malignant lesion.

The human crystalline lens offers natural protection by filtering UV and blue light. As an individual ages, the ability of the lens to naturally filter out blue light increases significantly [4,25]. In patients that undergo cataract surgery, the protection provided by the naturally yellowing crystalline lens is lost. Despite all the controversy about the use of blue light filtering lenses in humans, there is compelling evidence that visible blue light is potentially hazardous. Considering the projections for increases in life expectancy, patients are expected to live several years after cataract surgery and secondary lens implantation. Many years of cumulative exposure could be potentially dangerous especially in eyes harboring uveal nevi. It is estimated that between five and ten percent of the population have asymptomatic uveal nevi [26]. Therefore, the use of UV and blue light filtering IOLs could be considered a preventative measure against possible blue light induced malignant transformation of existing uveal nevi.

## Conclusion

In summary, we present evidence that blue light exposure can influence uveal melanoma cells and further substantiate the results of previous in vitro studies. Our data demonstrated a significant increase in uveal melanoma cellular proliferation after exposure to blue light. This data warrants further investigation assessing the efficacy of blue light filtering IOLs to slow the progression of uveal melanoma.

## Competing interests

The authors declare that they have no competing interests.

## Authors' contributions

SDC re-cultured the cell lines, ran all proliferation assays, and wrote the entire manuscript. SM organized the animal model, and oversaw all technical aspects of the model over the 8 week period. BFF performed weekly fundoscopic examinations, oversaw all gross and clinical histopathology for the entire model. CM was responsible for all blood extractions. JCM was responsible for all Ficoll-Paque processing throughout the model. EA performed all the immunohistochemistry. ANC was the second independent pathologist who graded all the immunohistochemistry. WWD was responsible for the design of the blue light setup. MNB Revised the entire manuscript.

## References

[B1] Demirci H, Shields CL, Shields JA, Honavar SG, Eagle RC (2002). Ring melanoma of the ciliary body: report on twenty-three patients. Retina (Philadelphia, Pa).

[B2] Singh A, Damato B, Murphree A, Perry J (2007). Clinical Ophthalmic Oncology.

[B3] McLean MJ, Foster WD, Zimmerman LE (1977). Prognostic factors in small malignant melanomas of choroid and ciliary body. Arch Ophthalmol.

[B4] Lerman S (1980). Radiant energy and the eye.

[B5] Albert DM, Jakobiec FA (1994). Principles and practice of ophthalmology: clinical practice.

[B6] Marshall JC, Gordon KD, McCauley CS, de Souza Filho JP, Burnier MN (2006). The effect of blue light exposure and use of intraocular lenses on human uveal melanoma cell lines. Melanoma research.

[B7] Manning WS, Greenlee PG, Norton JN (2004). Ocular melanoma in a Long Evans rat. Contemp Top Lab Anim Sci.

[B8] Csoma Z, Hencz P, Orvos H, Kemeny L, Dobozy A, Dosa-Racz E, Erdei Z, Bartusek D, Olah J (2007). Neonatal blue-light phototherapy could increase the risk of dysplastic nevus development. Pediatrics.

[B9] Saornil AM (2004). Iris Colour and Uveal Melanoma. CJO.

[B10] Singh AD, Rennie IG, Seregard S, Giblin M, McKenzie J (2004). Sunlight exposure and pathogenesis of uveal melanoma. Surv Ophthalmol.

[B11] King A, Gottlieb E, Brooks DG, Murphy MP, Dunaief JL (2004). Mitochondria-derived reactive oxygen species mediate blue light-induced death of retinal pigment epithelial cells. Photochem Photobiol.

[B12] Beatty S, Koh H, Phil M, Henson D, Boulton M (2000). The role of oxidative stress in the pathogenesis of age-related macular degeneration. Surv Ophthalmol.

[B13] Blanco PL, Marshall JC, Antecka E, Callejo SA, Souza Filho JP, Saraiva V, Burnier MN (2005). Characterization of ocular and metastatic uveal melanoma in an animal model. Invest Ophthalmol Vis Sci.

[B14] De Waard-Siebinga I, Blom DJ, Griffioen M, Schrier PI, Hoogendoorn E, Beverstock G, Danen EH, Jager MJ (1995). Establishment and characterization of an uveal-melanoma cell line. Int J Cancer.

[B15] Marshall JC, Caissie AL, Callejo SA, Antecka E, Burnier MN (2004). Cell proliferation profile of five human uveal melanoma cell lines of different metastatic potential. Pathobiology.

[B16] Steuhl KP, Rohrbach JM, Knorr M, Thiel HJ (1993). Significance, specificity, and ultrastructural localization of HMB-45 antigen in pigmented ocular tumors. Ophthalmology.

[B17] Burnier MN, McLean IW, Gamel JW (1991). Immunohistochemical evaluation of uveal melanocytic tumors. Expression of HMB-45, S-100 protein, and neuron-specific enolase. Cancer.

[B18] Skehan P, Storeng R, Scudiero D, Monks A, McMahon J, Vistica D, Warren JT, Bokesch H, Kenney S, Boyd MR (1990). New colorimetric cytotoxicity assay for anticancer-drug screening. J Natl Cancer Inst.

[B19] Shields CL (2008). The hunt for the secrets of uveal melanoma. Clin Experiment Ophthalmol.

[B20] Shah CP, Weis E, Lajous M, Shields JA, Shields CL (2005). Intermittent and chronic ultraviolet light exposure and uveal melanoma: a meta-analysis. Ophthalmology.

[B21] Smith JH, Padnick-Silver L, Newlin A, Rhodes K, Rubinstein WS (2007). Genetic study of familial uveal melanoma: association of uveal and cutaneous melanoma with cutaneous and ocular nevi. Ophthalmology.

[B22] Holly EA, Aston DA, Char DH, Kristiansen JJ, Ahn DK (1990). Uveal melanoma in relation to ultraviolet light exposure and host factors. Cancer Res.

[B23] Csoma Z, Hencz P, Orvos H, Kemeny L, Dobozy A, Dosa-Racz E, Erdei Z, Bartusek D, Olah J (2007). Neonatal blue-light phototherapy could increase the risk of dysplastic nevus development. Pediatrics.

[B24] Matichard E, Le Henanff A, Sanders A, Leguyadec J, Crickx B, Descamps V (2006). Effect of neonatal phototherapy on melanocytic nevus count in children. Arch Dermatol.

[B25] Ranjan M, Beedu SR (2006). Spectroscopic and biochemical correlations during the course of human lens aging. BMC ophthalmology.

[B26] Spencer WH, American Academy of Ophthalmology (1996). Ophthalmic pathology: an atlas and textbook. Ophthalmic pathology: an atlas and textbook.

